# The IFN-**γ** receptor promotes immune dysregulation and disease in STING gain-of-function mice

**DOI:** 10.1172/jci.insight.155250

**Published:** 2022-09-08

**Authors:** W. Alexander Stinson, Cathrine A. Miner, Fang R. Zhao, Annena Jane Lundgren, Subhajit Poddar, Jonathan J. Miner

**Affiliations:** 1 Department of Pathology and Immunology, Washington University School of Medicine, Saint Louis, Missouri, USA.; 2 Department of Medicine and Microbiology, University of Pennsylvania, Philadelphia, Pennsylvania, USA.; 3 Department of Medicine, Washington University School of Medicine, Saint Louis, Missouri, USA.

**Keywords:** Autoimmunity, Inflammation, Autoimmune diseases, Innate immunity, Monogenic diseases

## Abstract

STING gain-of-function mutations cause STING-associated vasculopathy with onset in infancy (SAVI) in humans, a disease characterized by spontaneous lung inflammation and fibrosis. Mice with STING gain-of-function mutations (SAVI mice) develop αβ T cell–dependent lung disease and also lack lymph nodes. Although SAVI has been regarded as a type I interferonopathy, the relative contributions of the three interferon receptors are incompletely understood. Here, we show that STING gain of function led to upregulation of IFN-γ–induced chemokines in the lungs of SAVI mice and that deletion of the type II IFN receptor (IFNGR1), but not the type I IFN receptor (IFNAR1) or type III IFN receptor (IFNλR1), ameliorated lung disease and restored lymph node development in SAVI mice. Furthermore, deletion of IFNGR1, but not IFNAR1 or IFNλR1, corrected the ratio of effector to Tregs in SAVI mice and in mixed bone marrow chimeric mice. Finally, cultured SAVI mouse macrophages were hyperresponsive to IFN-γ, but not IFN-β, in terms of *Cxcl9* upregulation and cell activation. These results demonstrate that IFNGR1 plays a major role in autoinflammation and immune dysregulation mediated by STING gain of function.

## Graphical abstract

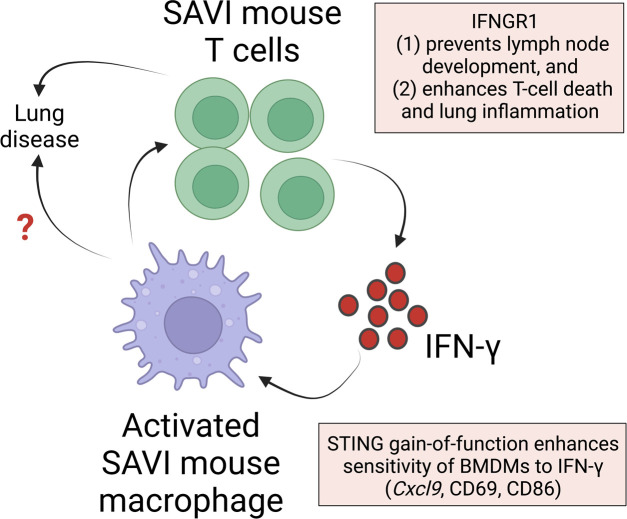

## Introduction

Stimulator of IFN genes (STING) plays a major role in regulating immunity against pathogens, but constitutive activation of STING causes inflammatory lung disease. STING is activated by the cyclic dinucleotide second messenger (2′3′ cyclic GMP-AMP [2′3′cGAMP]), which is produced by cGAMP synthase in response to double-stranded DNA in the cytosol (1, 2). Subsequent phosphorylation of STING by TANK-binding kinase-1 (TBK1) leads to the recruitment of IFN regulatory factor 3 (IRF3), a transcription factor that can induce expression of IFNs and upregulate hundreds of IFN-stimulated genes (ISGs) (3, 4). There are 3 broad classes of IFNs, which bind to distinct receptors (types I, II, and III). The contribution of each IFN receptor in SAVI mice is incompletely understood, so we set out to define the role of each IFN receptor in our mouse model of STING-associated lung disease (5–9).

Gain-of-function mutations in STING lead to autoinflammation in humans and mice (5, 10, 11). In humans, STING gain-of-function mutations cause a pediatric disease known as STING-associated vasculopathy with onset in infancy (SAVI). In mice with SAVI-associated STING gain-of-function mutations, inflammatory lung disease is associated with diminished numbers of T cells in the periphery, increased frequencies of cytokine-producing effector T cells, and reduced numbers of Tregs (5, 7, 12, 13). SAVI mice also lack lymph nodes, which may contribute to immune dysregulation (8). Although it was hypothesized that STING-associated lung disease would require the type I IFN receptor (IFNAR1) (10, 14), we and others demonstrated that IFNAR1 is dispensable for lung disease in SAVI mice (7, 12, 13). Because STING signaling can regulate production of type II (IFN-γ) and type III (IFNλ1–IFNλ4) IFNs (15–17), upregulation of ISGs in humans and mice with STING gain-of-function mutations (SAVI mice) might instead be attributable to the effects of multiple IFN receptors. Because STING signaling can regulate production of type II (IFN-γ) and type III (IFNλ1–IFNλ4) IFNs (15–17), upregulation of ISGs in humans and mice with STING gain-of-function mutations (SAVI mice) might instead be attributable to the effects of multiple IFN receptors. Furthermore, lung disease in SAVI mice is mediated by T cells (7), and STING signaling can regulate T cell proliferation and death (5, 15, 18), but which of the 3 IFN receptors might regulate T cell phenotypes had not been determined.

We hypothesized that autoinflammatory lung disease and T cell cytopenia in SAVI mice depends on 1 of the 3 IFN receptors. To test this hypothesis, we crossed our gain-of-function (SAVI) knockin mice to animals lacking IFNAR1, type II IFN receptor (IFNGR1), or type III IFN receptor (IFNλR1). We discovered that deletion of IFNGR1, but not IFNAR1 or IFNλR1, ameliorated inflammatory lung disease in SAVI mice. We show that macrophages from SAVI mice were hyperresponsive to IFNGR1. Deletion of IFNGR1, but not the other IFN receptors, completely restored lymph node development in SAVI mice. Amelioration of lung disease and restoration of lymph node development was associated with a near-complete restoration of a normal effector T cell–to-Treg ratio. Using coculture and mixed bone marrow chimeric mouse experiments, we demonstrated a cell-intrinsic contribution of IFNGR1 to SAVI mouse T cell survival. Thus, our results demonstrate that STING gain of function amplifies contributions of IFNGR1 in macrophages and T cells, including Tregs. Furthermore, our results indicate that a STING gain-of-function mutation causes a type II interferonopathy in mice.

## Results

**Figure 1:**
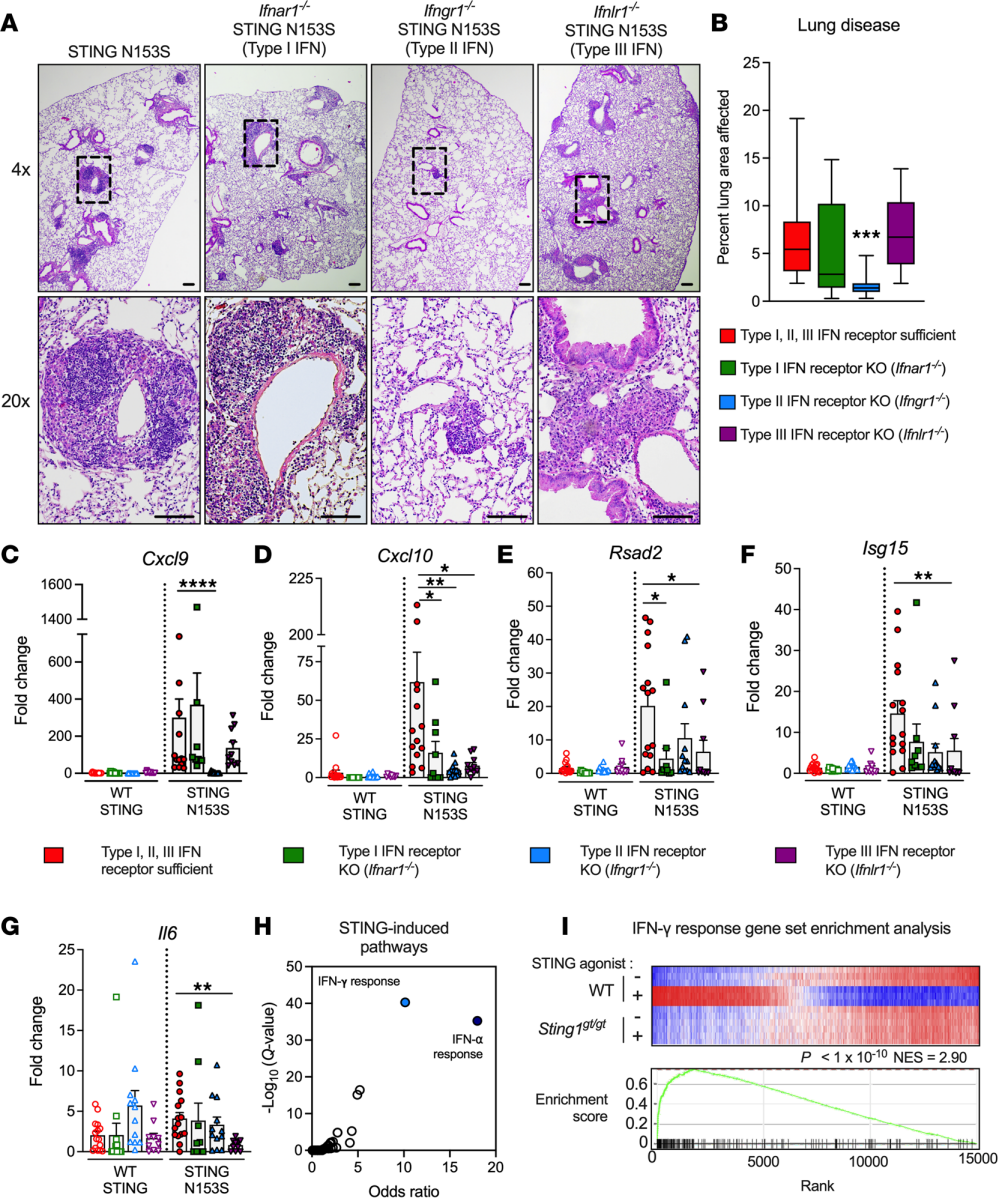
The type II IFN receptor promotes lung disease in SAVI mice. (**A**) Representative images of H&E-stained lung sections from 13- to 16-week-old STING N153S (SAVI) mice or the corresponding knockout (KO) animals lacking the indicated IFN receptor. Images are representative of *n* = 9–16 mice per group. The top row of images was taken under low magnification, and bottom row was taken under high magnification. Scale bars: 100 μm. Original magnification, ×4 (top); ×20 (bottom). (**B**) Quantitation of perivascular inflammation in the lungs from mice of indicated genotypes from **A**. Data are shown as the mean ± SEM. (**C–G**) ISG and cytokine expression in lungs from WT and SAVI animals of the indicated IFN receptor genotype. Data are shown as the mean ± SEM from *n* = 9–15 mice from 3 to 6 independent experiments. (**H**) Differentially upregulated transcriptional programs following STING activation in naive CD4^+^ T cells from the publicly available data set GSE100411 (20). Gene sets were obtained from the MSigDB Hallmark collection (45). (**I**) Enrichment plot of the hallmark IFN-γ response gene set following STING activation in naive CD4^+^ T cells (20). Fisher’s exact test and normalized enrichment score (NES) are included. Data in **B–G** were analyzed by Kruskal-Wallis test, comparing lung disease (**B**) or gene expression (**C–G**) in the lungs of the indicated knockout animals to values in the SAVI reference group. Statistical significance of differentially expressed gene sets in **I** was computed by Fisher’s exact test with Benjamini-Hochberg correction (FDR = 5%) using Enrichr (41–43), which reports the adjusted *P* value as a *Q* value. *P < 0.05, **P < 0.01, ***P < 0.001, ****P < 0.0001.

STING gain-of-function mutations that cause SAVI can upregulate the expression of ISGs and cause lung disease in humans and mice (5–7, 10). Although SAVI is generally thought to be a type I interferonopathy, there is considerable overlap in the gene expression signatures in the 3 IFN receptor pathways (19). To determine which IFN receptor promotes lung disease in SAVI mice, we histologically evaluated the lungs of age- and sex-matched SAVI mice, including SAVI mice lacking 1 of the 3 IFN receptors: IFNAR1, IFNGR1, or IFNλR1 (Figure 1, A and B, and Supplemental Figure 1A; supplemental material available online with this article; https://doi.org/10.1172/jci.insight.155250DS1). We observed that deletion of *Ifngr1* protects against autoinflammatory lung disease. In contrast, deletion of *Ifnar1* or *Ifnlr1* IFN receptors had no significant effect on lung disease in SAVI mice. However, certain features of disease were not corrected by deletion of any IFN receptor. For example, we observed that splenomegaly persists in SAVI mice, regardless of which IFN receptor is deleted (Supplemental Figure 1B), and that survival of *Ifngr1^–/–^* SAVI mice may be only slightly improved (Supplemental Figure 1C), suggesting additional IFN-independent or combinatorial IFN receptor–dependent effects.

*Cxcl9* encodes a chemokine that plays a major role in recruiting T cells to sites of inflammation, and its expression in the lungs of SAVI mice was diminished in the absence of IFNGR1 but not in other IFN receptor–knockout animals (Figure 1C). Expression levels of other ISGs, including *Cxcl10* and *Rsad2* but not *Isg15*, were diminished in the lungs of the type I and type III IFN receptor–knockout animals (Figure 1, D–F), suggesting that the other 3 IFN receptors may contribute to the inflammatory milieu, even when the histological appearance of the lungs is not affected. Additionally, expression of the gene encoding the proinflammatory cytokine IL-6 was reduced only in mice lacking IFNλR1 (Figure 1G). These results suggest that all 3 IFN receptors may regulate gene expression in the lungs of SAVI mice, although IFNGR1 had the greatest effect on expression of chemokines known to recruit T cells. Because STING-associated lung disease is partially mediated by αβ T cells (7), we reasoned that STING signaling may influence type II IFN–related pathways. Indeed, our pathway analysis of a previously published gene expression data set (20) suggests that STING activation in murine CD4^+^ T cells may be associated with induction of both type II (IFN-γ) and type I (IFN-α) pathways (Figure 1, H and I), further indicating a potential link between STING signaling and type II IFN.

**Figure 2:**
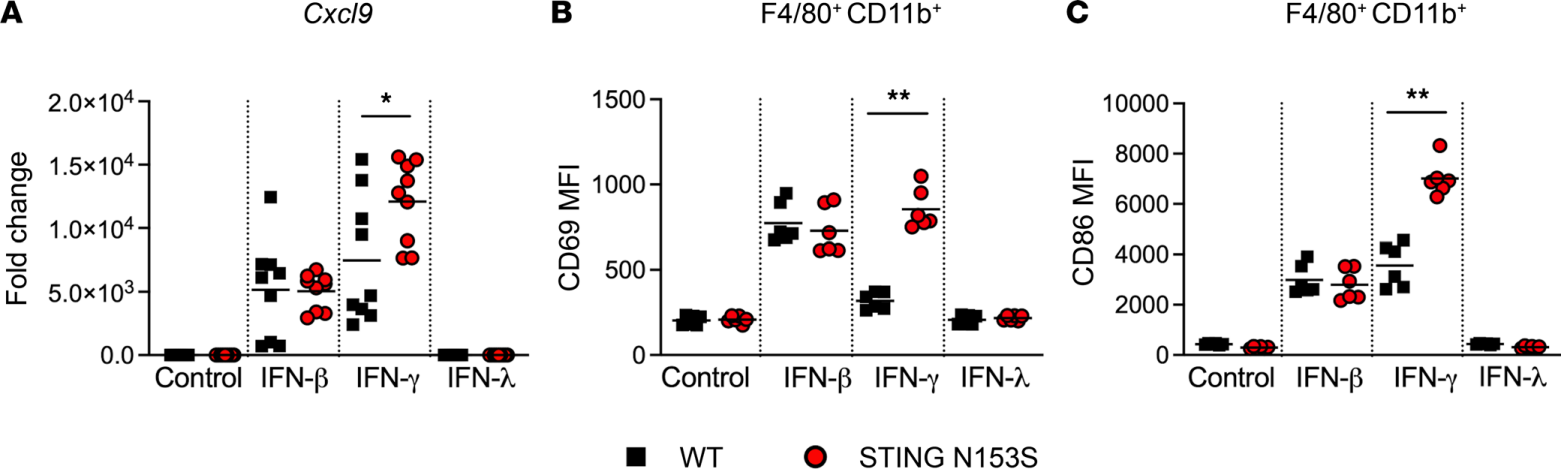
IFN-γ enhances *Cxcl9* expression and upregulates activation markers in STING gain-of-function bone marrow–derived macrophages. (**A**) *Cxcl9* expression in WT and STING gain-of-function mouse bone marrow–derived macrophages was measured by qRT-PCR following treatment with IFN-β (100 U/mL), IFN-γ (10 ng/mL), or IFN-λ (100 ng/mL) for 4 hours. Data represent the mean of *n* = 9 samples pooled from 3 independent experiments. (**B** and **C**) Mean fluorescent intensity of cell surface activation markers on macrophages following treatment with type I, II, or III IFN for 24 hours. Data represent the mean of *n* = 6 samples pooled from 2 independent experiments. Data in **A–C** were analyzed by Mann-Whitney test. *P < 0.05, **P < 0.01.

To further assess whether STING gain of function enhances chemokine induction in response to IFN-γ, we quantitated *Cxcl9* expression in bone marrow–derived macrophages (BMDMs) after treatment with types I, II, or III IFN. We found that IFN-γ caused SAVI mouse BMDMs to upregulate *Cxcl9* more robustly than in WT BMDMs. In contrast, IFN-β equivalently induced *Cxcl9* expression in WT and SAVI mouse macrophages, and IFN-λ had no effect on *Cxcl9* expression (Figure 2A). We also observed enhanced IFN-γ–induced upregulation of cell surface activation markers such as CD69 and CD86 in SAVI mouse macrophages (Figure 2, B and C). This suggests that STING gain of function and IFNGR1 cooperate to promote macrophage activation in SAVI mice.

**Figure 3:**
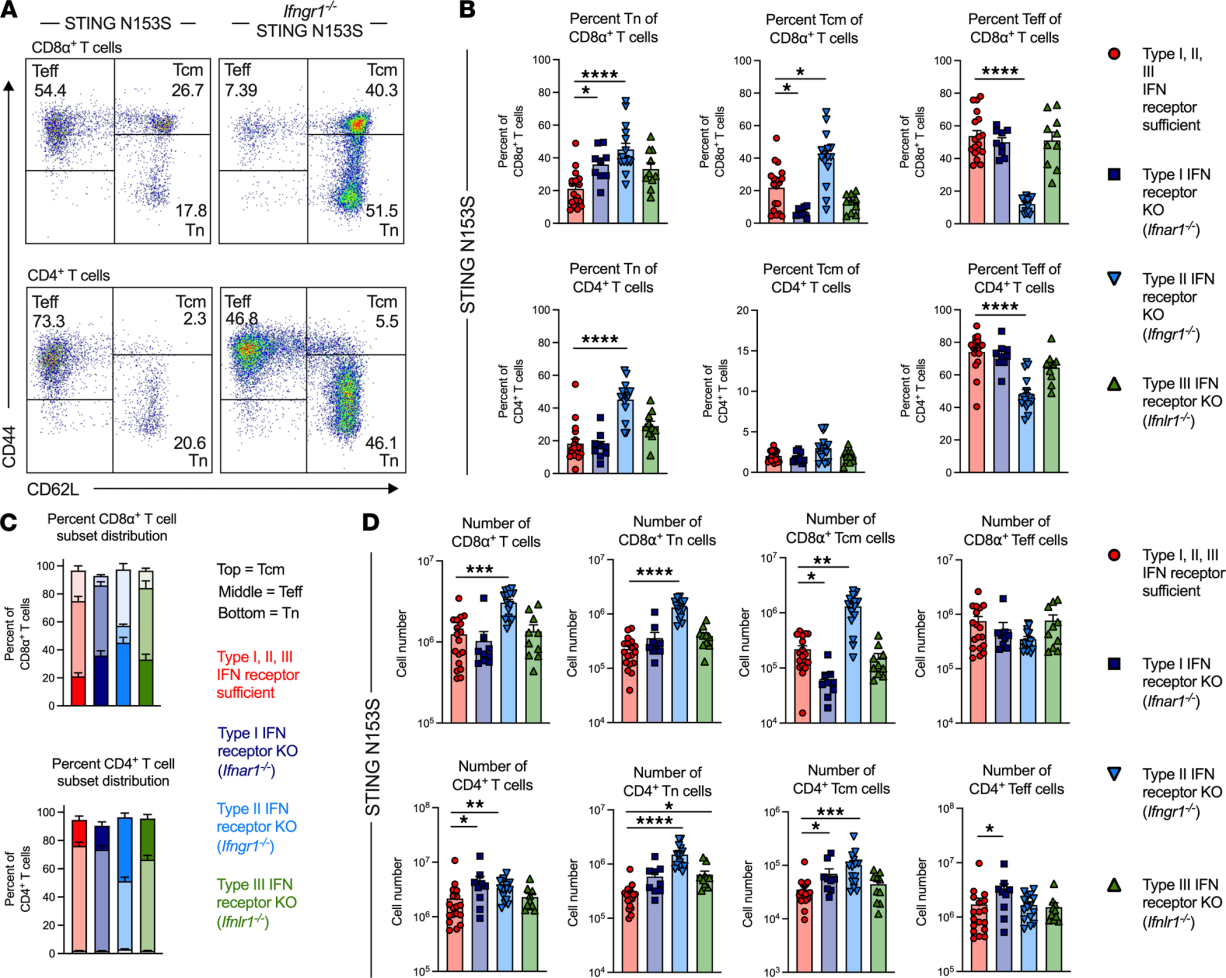
The type II IFN receptor, IFNGR1, regulates frequencies, numbers, and subsets of splenic T cells in SAVI mice. (**A**) Representative FACS plots of CD44^lo^CD62L^hi^ naive (Tn), CD44^hi^CD62L^hi^ central memory (Tcm), and CD44^hi^CD62L^lo^ effector memory (Teff) CD8α^+^ (top) and CD4^+^ (bottom) T cells from STING N153S (SAVI) and *Ifngr1*^–/–^ SAVI mice. (**B**) Frequencies of splenic CD8α^+^ and CD4^+^ Tn, Tcm, and Teff cells in SAVI animals or those lacking the indicated IFN receptor. Frequencies are of total CD8α^+^ or CD4^+^ T cells. (**C**) Comparison of subset distributions of CD8α^+^ T cells and CD4^+^ T cells in SAVI mice with functioning type I, II, and III IFN receptors or those lacking the indicated IFN receptor. (**D**) Numbers of total splenic CD8α^+^ or CD4^+^ T cells, as well as CD8α^+^ or CD4^+^ Tn, Tcm, and Teff subsets, in SAVI mice or those lacking the indicated IFN receptor. Data in **B–D** represent the mean ± SEM of *n =* 9–18 mice per genotype pooled from 3 to 6 independent experiments. Data were analyzed by Kruskal-Wallis test. *P < 0.05, **P < 0.01, ***P < 0.001, ****P < 0.0001.

**Figure 4:**
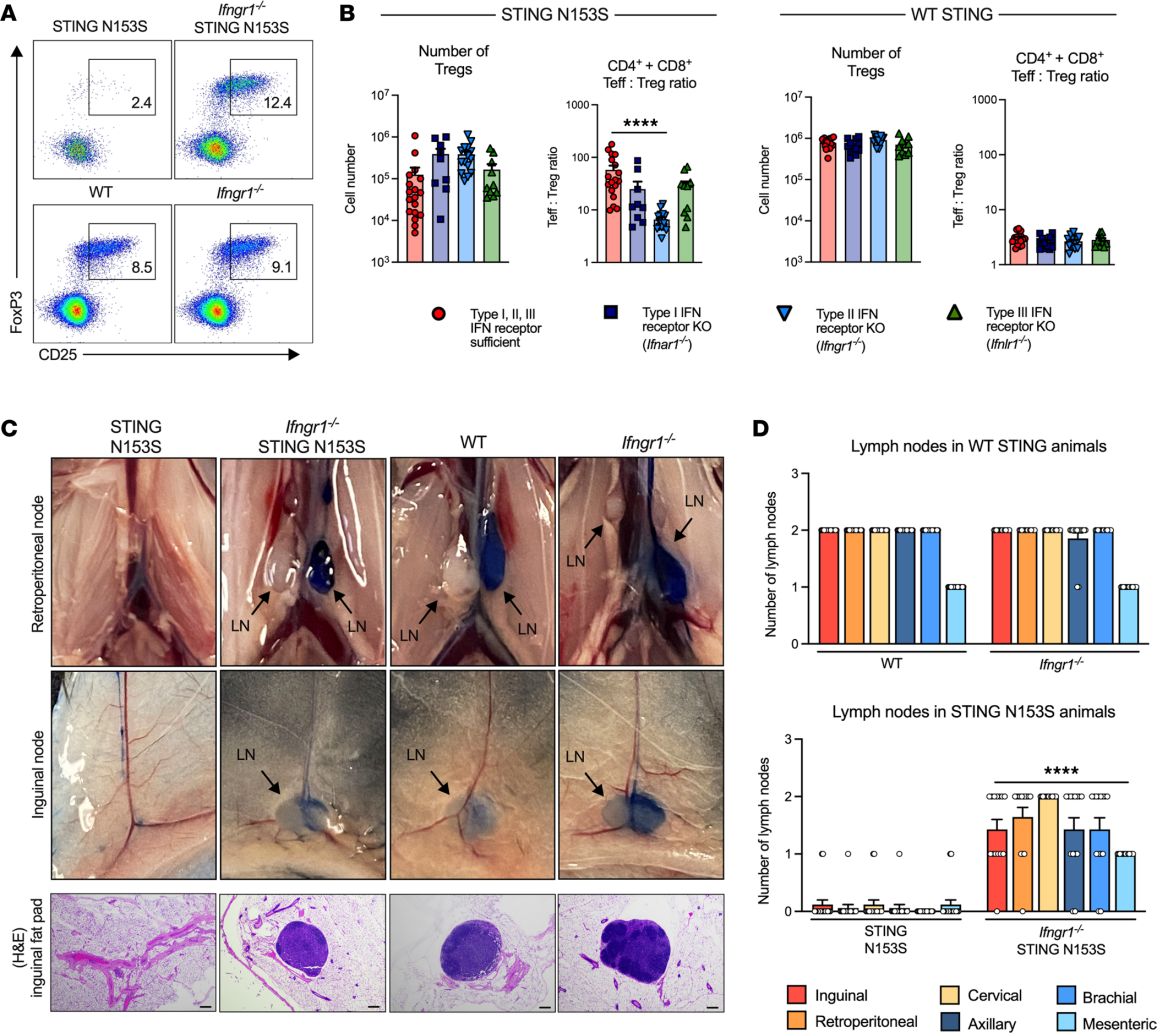
Deletion of IFNGR1 partially rescues Treg and lymph node deficiencies in SAVI mice. (**A**) Representative FACS plots depicting gating strategy to define splenic CD25^+^ FoxP3^+^ Tregs from STING N153S (SAVI) and *Ifngr1^–/–^* SAVI mice (top) and WT and *Ifngr1^–/–^* animals (bottom). Cells were pregated on CD3ε^+^NK1.1^–^CD4^+^CD8^–^ T cells. (**B**) Numbers of splenic Tregs and the ratio of the number of splenic CD4^+^ and CD8α^+^ Teff cells to Tregs in 13- to 16-week-old SAVI mice (left) or their WT STING-expressing littermates (right), including corresponding knockout animals lacking the indicated IFN receptor. Data are shown as the mean ± SEM of *n =* 9–18 mice per genotype pooled from 3 to 6 independent experiments. (**C**) Images of retroperitoneal and inguinal lymph nodes from SAVI mice, *Ifngr1^–/–^* SAVI mice, WT mice, and *Ifngr1^–/–^* mice, 15 minutes after injection of Evans blue dye. Representative sections of H&E-stained inguinal fat pads further displays lymph node development. Scale bar: 100 µm. (**D**) Number of lymph nodes in WT mice versus *Ifngr1^–/–^* mice and SAVI mice versus *Ifngr1^–/–^* SAVI mice. Data are shown as the mean ± SEM of *n =* 14–17 mice per genotype. Data in **B** were analyzed by Kruskal-Wallis test. Data in **D** were analyzed by Mann-Whitney test. ****P < 0.0001.

Because T cells play a major role in lung disease in SAVI mice (7), we reasoned that IFNGR1 may also have direct effects on T cells. SAVI mice have T cell cytopenia as well as major alterations in the number or frequency of naive and effector T cells, including diminished numbers of total T cells and increased percentages of effector T cells (7). We found that the frequency and number of total, naive, and central memory CD4^+^ and CD8α^+^ T cells was greater in *Ifngr1^–/–^* SAVI animals compared with that in SAVI mice that express IFNGR1 (Figure 3). In contrast, deletion of IFNGR1 in animals expressing WT STING had no effect on any of the T cell populations analyzed (Supplemental Figure 2, A–D), indicating that the detrimental effects of IFNGR1 on T cells occur specifically in the context of mutant STING. In further support of a major role for IFNGR1-induced pathology in STING gain-of-function T cells, deletion of IFNGR1, but not IFNAR1 or IFNλR1, in SAVI mice led to a large increase in the number of Tregs and corrected the ratio of effector T cells to Tregs (Figure 4, A and B). In contrast, deletion of IFNGR1 had no effect on myeloid cell numbers in SAVI animals (Supplemental Figure 3, A and B). This is consistent with the idea that IFNGR1 expression in SAVI mice affects survival and proliferation of T cells without affecting numbers of myeloid cells.

SAVI mice lack lymph nodes due to effects of the STING mutant that are independent of T and B cells (8). Remarkably, deletion of IFNGR1, but not IFNAR1 and IFNλR1, restored lymph node development in SAVI mice (Figure 4, C and D). This underscores the major role of IFNGR1 in disease pathogenesis in SAVI mice and suggests additional interactions between IFNGR1 and STING beyond those in T cells and macrophages.

**Figure 5:**
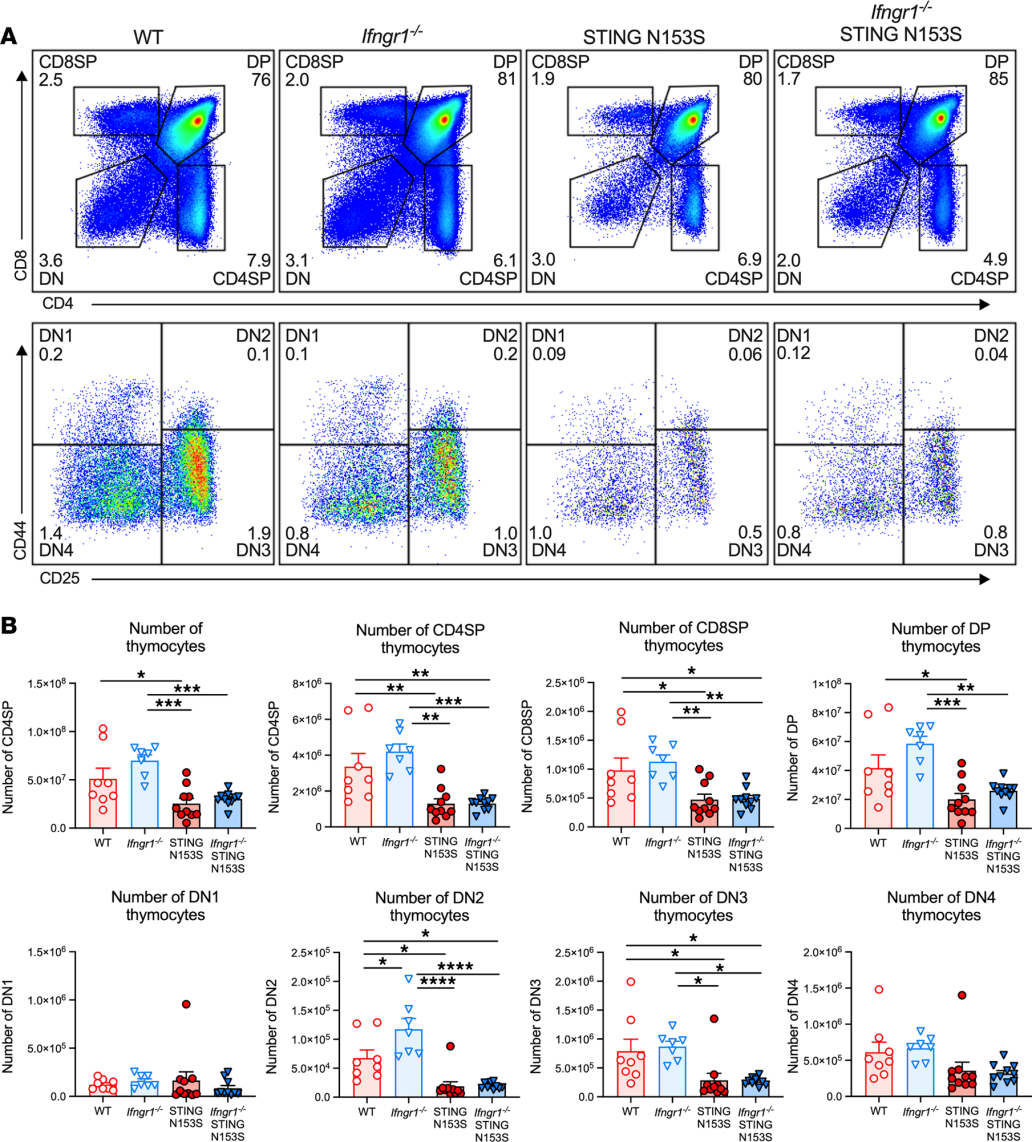
IFNGR1 deletion does not alter thymocyte frequencies and numbers in SAVI mice. (**A**) Representative FACS plots depicting gating strategy to define CD8α^+^ single-positive (CD8SP), CD4^+^CD8α^+^ double-positive (DP), CD4^+^ single-positive (CD4SP), and CD4^–^CD8α^–^ double-negative (DN) thymocytes from WT, *Ifngr1^–/–^*, STING N153S (SAVI), or *Ifngr1^–/–^* SAVI animals. Depicted thymocytes were pregated on CD45^+^CD11b^–^CD19^–^NK1.1^–^ cells. Representative FACS plots depicting gating strategy to define DN thymocyte subsets, CD44^+^CD25^–^ (DN1), CD44^+^CD25^+^ (DN2), CD44^–^CD25^+^ (DN3), and CD44^–^CD25^–^ (DN4), in mice of the indicated genotypes. (**B**) Numbers of total CD45^+^, DP, CD4SP, CD8SP, and DN1, DN2, DN3, DN4 thymocytes in 8- to 12-week-old WT, *Ifngr1^–/–^*, SAVI, or *Ifngr1^–/–^* SAVI animals. Data represent the mean of *n* = 8–10 mice per genotype pooled from 3 to 4 independent experiments. Results were analyzed by Kruskal-Wallis test. *P < 0.05, **P < 0.01, ***P < 0.001, ****P < 0.0001.

We previously found that SAVI mice exhibit diminished numbers of thymocytes at all stages of T cell development, and we confirmed that this effect was intrinsic to the hematopoietic compartment based on studies of mixed bone marrow chimeric mice (5, 7). To determine whether IFNGR1 regulates T cell development in SAVI mice, we performed flow cytometry to quantitate the numbers of thymocyte subsets in WT and SAVI animals. We found that the absence of IFNGR1 did not alter WT or SAVI mouse thymocyte numbers at any developmental stage in the thymus (Figure 5). Thus, IFNGR1 likely regulates survival and/or proliferation of SAVI T cells in the periphery.

**Figure 6:**
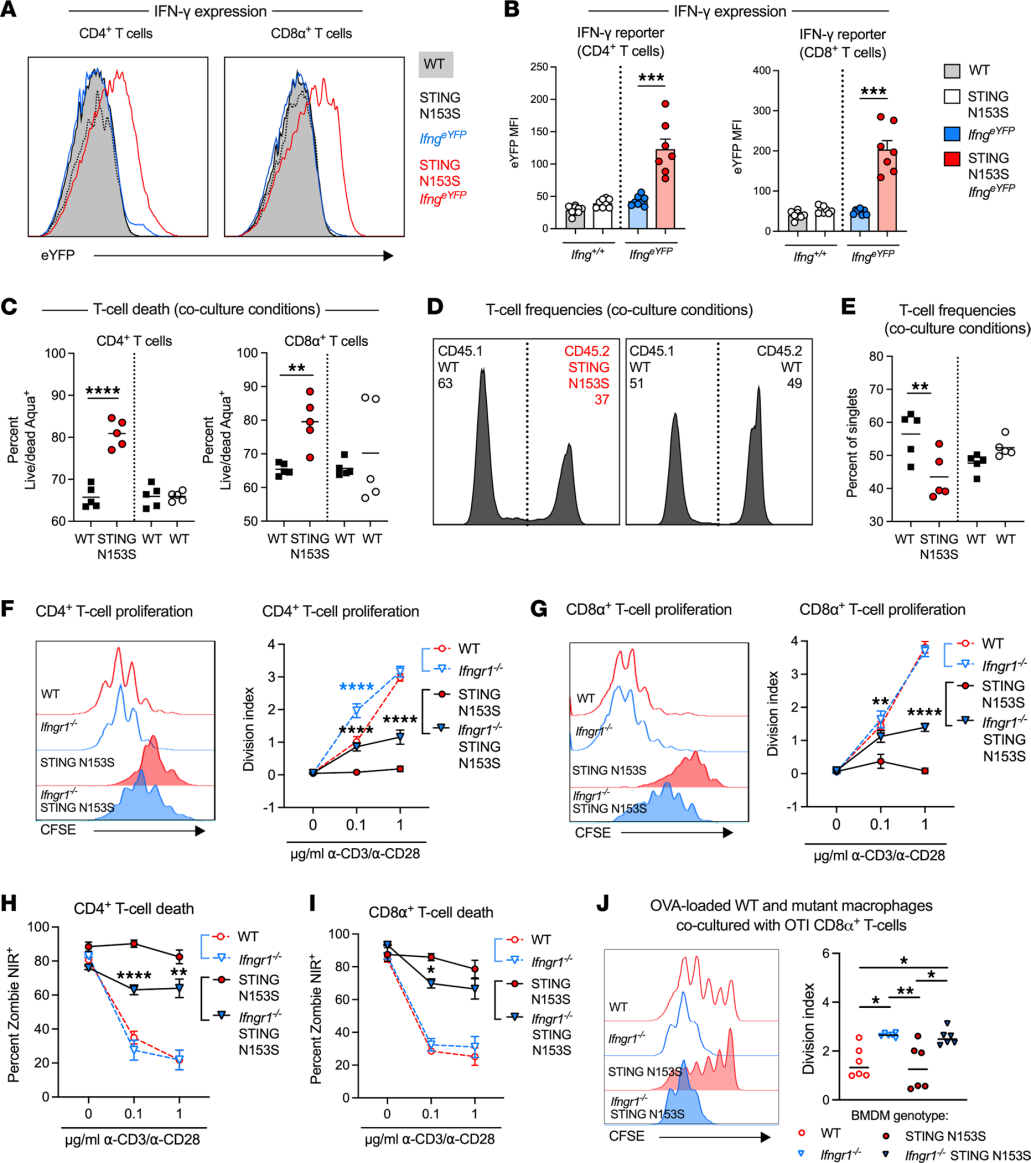
IFNGR1 partially mediates STING-associated T cell proliferation and survival defects in cell culture. (**A**) Representative FACS plots showing eYFP expression in splenic CD4^+^ and CD8α^+^ T cells from WT and STING N153S (SAVI) IFN-γ reporter (*Ifng^eYFP^*) and nonreporter (*Ifng^+/+^*) mice. (**B**) CD4^+^ and CD8α^+^ T cell eYFP mean fluorescence intensity (MFI) in animals from **A**. Data represent the mean of *n =* 7–9 mice pooled from 2 independent experiments. (**C**) Percentage of T cells after 24-hour coculture that stained positively for the viability dye LIVE/DEAD Aqua. (**D**) Representative FACS plots depicting frequency of T cells isolated from mice of the indicated genotype following 24 hours in coculture. (**E**) Percentage distribution of isolated WT or SAVI T cells following 24-hour coculture. T cells were isolated from CD45.1 WT, CD45.2 SAVI, or CD45.2 WT animals, and data represent the mean cellular frequency of *n* = 5 samples pooled from 2 independent experiments. (**F** and **G**) Representative FACS plots depicting dilution of CFSE in and the resulting division indices ± SEM of CD4^+^ T cells (**F**) or CD8α^+^ T cells (**G**) following 3-day mock or α-CD3ε/α-CD28 stimulation of bulk splenocytes isolated from *n* = 5–6 mice of the indicated genotypes. (**H** and **I**) Percentage cell death of CD4^+^ (**H**) and CD8α^+^ (**I**) T cells following 3-day mock or α-CD3ε/α-CD28 stimulation of bulk splenocytes isolated from *n* = 5–6 mice of the indicated genotypes. (**F–I**) Data represent the mean pooled 3 independent experiments. (**J**) WT STING-expressing OTI CD8α^+^ T cell proliferation in response to WT and SAVI bone marrow–derived macrophages (BMDMs) loaded with OVA peptide. BMDMs were harvested loaded with OVA peptide under conditions of LPS stimulation, and then cultured with WT STING-expressing OTI T cells for 3 days. Data represent the mean of *n* = 6 samples pooled from 2 independent experiments. Data were analyzed by Student’s *t* test (**B**); 1-way ANOVA (**C**, **E**, and **J**); 2-way ANOVA (**F–I**). *P < 0.05, **P < 0.01, ***P < 0.001, ****P < 0.0001.

To further explore the relationship between STING gain of function and IFN-γ expression, we crossed our SAVI mice to homozygous IFN-γ–internal ribosome entry site–enhanced yellow fluorescent protein (IFN-γ–IRES-eYFP) reporter (*Ifng*^eYFP^) animals. We found that SAVI mouse CD4^+^ and CD8α^+^ T cells exhibited significant upregulation of the IFN-γ reporter (Figure 6, A and B), as well as IFN-γ stained with a monoclonal antibody (Supplemental Figure 4, A–F). Upregulation of IFN-γ was not observed in other hematopoietic cell types, including NK cells and myeloid cells (Supplemental Figure 5, A and B). We reasoned that IFN-γ, or perhaps another soluble factor secreted by SAVI mouse T cells, might affect survival of neighboring T cells in a paracrine fashion. To test this hypothesis, we cocultured CD45.2 WT T cells with congenic CD45.1 SAVI or WT T cells and examined cell death 24 hours later by cellular uptake of a viability dye. Consistent with our previously published data (21), we found that SAVI T cells spontaneously die more frequently than WT T cells in a competitive coculture system (Figure 6, C–E). Moreover, we found that death of WT T cells was not affected by the presence of cocultured congenic STING N153S or WT T cells (Figure 6, C–E), indicating a cell-intrinsic effect of the STING gain-of-function mutant on T lymphocytes.

In T cells, activation of STING has cell-intrinsic antiproliferative activity (18, 21). Thus, we hypothesized that IFNGR1 may also play a role in STING-mediated inhibition of T cell proliferation. To test this hypothesis, we performed in vitro CD3ε/CD28 costimulation experiments using splenocytes isolated from WT, *Ifngr1^–/–^*, SAVI, and *Ifngr1^–/–^* SAVI mice. We found that deletion of *Ifngr1* in SAVI CD4^+^ and CD8α^+^ T cells partially corrected the SAVI-associated defect in cellular expansion following CD3ε/CD28 cross-linking (Figure 6, F and G). Indeed, SAVI mouse T cells were almost entirely incapable of proliferation when IFNGR1 expression was intact, although this may be explained by enhanced activation-induced cell death, which was diminished in the absence of IFNGR1 (Figure 6, H and I). Comparatively, WT and *Ifngr1^–/–^* T cells displayed robust, equivalent proliferation (Figure 6, F and G), suggesting that the effects of IFNGR1 on T cell proliferation occur only in cells from SAVI mice. However, SAVI mouse T cells underwent spontaneous death regardless of whether they were treated with type I, type II, or type III IFN (Supplemental Figure 6, A and B). Additionally, blockade of IFN-γ with a neutralizing antibody had no effect on survival of T cells from WT and SAVI mice in cell culture, suggesting that effects of IFN-γ on T cells may occur via an indirect mechanism or through interactions with another cell type (Supplemental Figure 7).

We hypothesized that IFNGR1 may have distinct effects on WT and SAVI mouse macrophages in terms of antigen presentation and that this might partially explain the effects of IFNGR1 deletion. To test this hypothesis, we performed T cell coculture experiments with WT and SAVI mouse littermate BMDMs and also with *Ifngr1^–/–^* and *Ifngr1^–/–^* SAVI mouse BMDMs. WT STING-expressing OTI T cell receptor–transgenic T cells, specific for a chicken ovalbumin peptide presented in the context of H2b, proliferated similarly in response to the ovalbumin peptide presented by WT and SAVI mouse BMDMs (Figure 6J). Furthermore, both *Ifngr1^–/–^* and *Ifngr1^–/–^* SAVI mouse BMDMs induced more robust proliferation of OTI T cells than IFNGR1-sufficient controls (Figure 6J). Collectively, our results shown in Figures 2 and 6 indicate a multifaceted role of IFNGR1 in modulation of macrophage function, including both STING-related and unrelated effects of IFNGR1.

**Figure 7:**
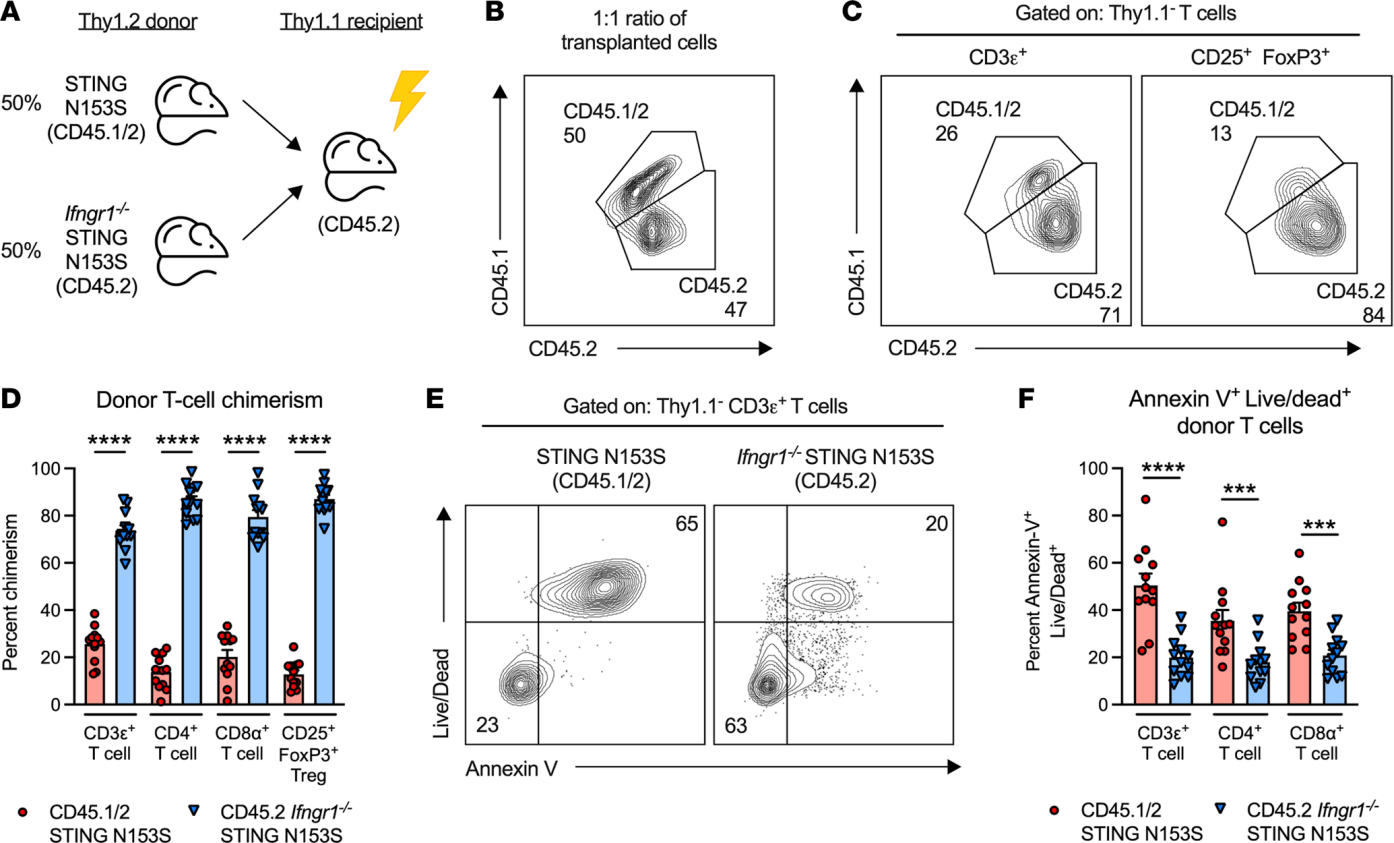
SAVI mouse T cells lacking the type II IFN receptor have a survival advantage in mixed bone marrow chimeric mice. (**A**) Diagram of the strategy used to generate Thy1.1 mixed bone marrow chimeric mice. (**B**) Representative FACS plot of the CD45.1/2 STING N153S (SAVI) and CD45.2 *Ifngr1^–/–^* SAVI mouse bone marrow cells, which were transferred at an approximately 1:1 ratio. (**C**) Representative FACS plots depicting donor (Thy1.1^–^) cell chimerism within circulating CD3ε^+^ T cells and CD25^+^ FoxP3^+^ Tregs in the irradiated Thy1.1^+^ animals. (**D**) Percentages of circulating CD45.1/2 SAVI and CD45.2 *Ifngr1^–/–^* STING N153S CD3ε^+^, CD4^+^, CD8α^+^ T cells and CD25^+^FoxP3^+^ Tregs in irradiated Thy1.1^+^ animals. (**E**) Representative FACS plots depicting the distribution of late apoptotic donor CD3ε^+^ T cells (defined as annexin V^+^ LIVE/DEAD^+^) in the irradiated Thy1.1^+^ chimeric mice shown. Shown are CD45.1/2 SAVI (left) and CD45.2 *Ifngr1^–/–^* SAVI T cells (right). (**F**) Percentage of annexin V^+^ LIVE/DEAD^+^ CD45.1/2 SAVI and CD45.2 *Ifngr1^–/–^* SAVI CD3ε^+^, CD4^+^, and CD8α^+^ T cells. Data in **C**, **E**, and **F** represent the mean ± SEM of the indicated T cell populations isolated from the spleens of *n* = 12 Thy1.1^+^ chimeric mice from 2 independent experiments. Data were analyzed by Mann-Whitney test. ***P < 0.001, ****P < 0.0001.

Because deletion of IFNGR1 restores lymph node development, affects chemokine production, and alters macrophage function, we reasoned that a mixed bone marrow chimeric mouse model would allow us to more definitively test whether effects of IFNGR1 deletion on T cells might explain partial resistance to STING-induced T cell death in vivo. We generated mixed bone marrow chimeric animals by transplanting a 1:1 mixture of IFNGR1-sufficient and IFNGR1-deficient SAVI mouse bone marrow into irradiated Thy1.1 recipient animals (Figure 7, A and B). Utilizing Thy1.1 recipient mice allowed us to examine effects of IFNGR1 on SAVI mouse T cell frequencies and survival in recipients that have intact lymph nodes. Six weeks after engraftment, we found that the majority of donor CD3ε^+^, CD4^+^, and CD8α^+^ T cells and CD25^+^ FoxP3^+^ Tregs were derived from *Ifngr1^–/–^* SAVI mouse bone marrow (Figure 7, C and D). In mixed bone marrow chimeric animals, IFNGR1 expression was associated with greater frequency of T cell death, suggesting a hematopoietic compartment-intrinsic effect of IFNGR1 on STING-associated T cell death in vivo (Figure 7, E and F). In contrast, mixed bone marrow chimeric mice engrafted with WT or *Ifngr1^–/–^* bone marrow exhibited similar frequencies of T cells, suggesting that IFNGR1-mediated regulation occurs specifically in SAVI mice (Supplemental Figure 8, A–C). Collectively, our results demonstrate immunological crosstalk between STING gain of function and IFNGR1 in a model of lung inflammation, during regulation of myeloid cell and T cell phenotypes and in the context of lymph node development. These results underscore the importance of IFNGR1 in STING-associated autoinflammation and will lead to future studies that define the cell type–specific contributions of IFN-γ and its receptor (IFNGR1) in the context of immune dysregulation and pathology mediated by STING gain of function.

## Discussion

Although it has been suggested that IFNAR1 mediates STING-associated autoinflammation (10, 14), we discovered an unexpected role for IFNGR1 in regulating lung disease and T cell and macrophage dysfunction in SAVI mice that have a STING gain-of-function mutation. In contrast, deletion of IFNAR1 or IFNλR1s did not ameliorate lung disease or affect T cell and macrophage phenotypes in SAVI animals. Thus, lung disease and immune dysregulation mediated by STING gain of function represent a type II interferonopathy in mice. Nevertheless, we cannot exclude more subtle contributions of IFNAR1 and IFNλR1s in this model or in other SAVI-associated phenotypes or models.

Multiple animal models and human diseases are linked to constitutive STING signaling (22–29), but disease phenotypes associated with STING activation are variable and depend on the specific genetic defect. For example, even though TREX1 deficiency causes activation of STING, the disease phenotypes of TREX1-knockout animals are entirely distinct from those of SAVI mice (5). Whereas SAVI mice exhibit inflammatory lung disease, T cell cytopenia, and immunodeficiency (5, 6), TREX1-knockout animals develop lupus-like disease characterized by cardiac inflammation without lung disease (5, 22). Even though ISGs and type I IFN are upregulated in both TREX1-knockout mice and SAVI-knockin animals (5, 22), deletion of IFNAR1 is protective only in TREX1-knockout animals and not in SAVI mice (7, 22). Our discoveries indicate that STING-associated lung disease and T cell phenotypes are indeed IFN related but unexpectedly mediated by IFNGR1, rather than by IFNAR1 or IFNλR1. Thus, whereas TREX1 deficiency causes a type I interferonopathy in mice (22), STING gain of function results in a type II interferonopathy, at least with respect to lung disease and T cell phenotypes in mice. The differences might be explained, in part, by the fact that TREX1 deficiency causes more profound induction of ISGs than STING gain of function (5).

In both TREX1-knockout and SAVI mouse models, T cells play a major role in disease pathogenesis (7). STING signaling in T cells is well established as a trigger of cell death and to be antiproliferative (15, 18, 20), but the contributions of each IFN receptor in SAVI T cell biology had not previously been established to our knowledge. We found that IFNGR1 signaling, and not IFNAR1 or IFNλR1 signaling, can regulate T cell survival and proliferation in SAVI mice. The effects of IFNGR1 signaling on SAVI mouse T cells likely occur preferentially in the periphery, because only peripheral T cells, and not thymocytes, were affected by deletion of IFNGR1. Furthermore, our mixed bone marrow chimeric mouse experiments suggest that the effects of IFNGR1 on T cells are intrinsic to the hematopoietic compartment and specific to the STING mutant.

In addition to hematopoietic compartment-intrinsic effects of IFNGR1 signaling, IFNGR1 may regulate T cell survival and proliferation indirectly via effects on other cells of the hematopoietic compartment (30–32). Indeed, we found that macrophages from SAVI mice were more sensitive to IFN-γ in terms of chemokine upregulation as well as expression of activation markers. Another potentially indirect effect of IFNGR1 is demonstrated by the fact that its absence restores lymph node development in SAVI mice. Indeed, to more completely define the multiple direct and indirect effects of IFNGR1, cell type–specific knockout mouse studies will be necessary. Nevertheless, our mixed bone marrow chimeric mouse studies revealed hematopoietic cell–intrinsic effects of IFNGR1 on SAVI mouse T cells, including numbers of Tregs. It seems likely that there are direct and indirect contributions of IFNGR1 to T cell phenotypes as well as in lymph node development. For instance, stromal cells of secondary lymphoid organs can regulate T cell responses through the antiproliferative effects of IFNGR1-induced nitric oxide production (33). Future studies involving the cell type–specific deletion of *Ifngr1* will determine in which cells IFNGR1 expression is necessary and sufficient to regulate lymph node development, lung disease, and T cell survival in SAVI mice. For example, the STING gain-of-function mutant interferes with LTi cell development, and it is possible that IFNGR1 regulates either LTi cell development or, alternatively, the recruitment of LTi cells to lymph node anlagen in SAVI mice.

Janus kinases (JAKs) have been targeted therapeutically in SAVI (10, 14, 34), and IFN receptors have preferential requirements for specific JAK proteins, including JAK1, JAK2, and TYK2 (35). Understanding which cytokine receptors are involved in specific features of SAVI pathogenesis could eventually become therapeutically relevant for the treatment of many forms of STING-associated autoinflammation in humans, because different JAK inhibitors exhibit some selectivity for specific kinases. However, it is important to note that some clinical phenotypes in humans with SAVI, such as pulmonary fibrosis, are not recapitulated in the mouse model. Nevertheless, our findings in SAVI mice may prompt additional studies in humans or nonhuman primates to determine the relevance of IFNGR1 and its downstream effectors in STING-associated inflammation.

## Methods

### Mice.

All mice were bred and cohoused in specific pathogen–free facilities at the University of Pennsylvania Perelman School of Medicine and at Washington University. Heterozygous STING N153S (SAVI) mice were generated as previously described (5). *Ifnar1^–/–^* STING N153S (7) and heterozygous *Ptprc^a/b^* (CD45.1/2) STING N153S mice were also previously generated (8). STING N153S animals were bred to congenic *Ifngr1^–/–^* (36), *Ifnlr1^–/–^* (37), or homozygous IFN-γ IRES enhanced YFP reporter mice (*Ifng^eYFP^* mice) (38). *Ifngr1^–/–^* and IFN-γ reporter animals were purchased from The Jackson Laboratory (003288 and 017581, respectively). *Ifnlr1^–/–^* mice were gifts from Megan Baldridge (Washington University). For antigen-presentation assays, OTI mice were purchased from The Jackson Laboratory (003831). For mixed bone marrow chimera studies, 6-week-old syngeneic *Thy1^a^* (Thy1.1) C57BL/6J mice were purchased from The Jackson Laboratory (000406). In all experiments, heterozygous STING N153S animals were compared with cohoused WT STING littermate control animals. For histological assessment of lungs, FACS analysis, and ISG expression, 13- to 16-week-old mice were euthanized, and their organs were harvested and processed for downstream applications. For T cell proliferation studies or bone marrow isolation, mice aged 4–6 weeks were studied.

### Histology and lung disease quantitation.

Dissected spleens, livers, and lungs were fixed overnight in 4% paraformaldehyde at room temperature (~22°C) and washed in 70% ethanol before paraffin embedding and sectioning. To facilitate serial sectioning of inguinal fat pads through the inguinal node, resected fat pads were pinned flat and stained overnight in Davidson’s fixative (1:3:5:1 ratio of neutral-buffered formalin, 95% ethanol, distilled H_2_O, and glacial acetic acid), rinsed in 70% ethanol, and encased in SeaPlaque low-melting temperature agarose (Lonza, 50101) to preserve orientation prior to paraffin embedding. Tissue sections (3 μm thick) were stained with H&E, and slides were imaged on a Nikon Eclipse Ci-L microscope equipped with a DS-Fi3 camera, using NIS Elements Basic Research software (Nikon). Quantitation of lung inflammation was performed with ImageJ (NIH) software by a histologist blinded to the identity of specimens as previously described (7). The percentage of lung area affected was determined as the number of pixels within lung lesions divided by the total number of pixels in lung tissue, excluding large airway spaces.

### RNA-sequencing analysis.

Gene expression from the publicly available WT and *Sting1^gt/gt^* CD4^+^ T cell data set (GSE100411) (20) was analyzed using the Phantasus online service (https://artyomovlab.wustl.edu/phantasus/) (39). Transcripts associated with nonannotated gene IDs were removed, and read counts were normalized by log_2_(1 + *x*) transformation. The top 1.5 × 10^4^ genes rank ordered by mean expression were analyzed for differential expression using the limma package (40). Pathway analysis using the MSigDB hallmark (H) gene set collection was performed on genes with log_2_ fold change greater than 1 using the online tool, Enrichr (https://maayanlab.cloud/Enrichr/) (41, 42). Statistical significance of the upregulated MSigDB hallmark gene sets was determined in Enrichr (41–43) as described below. Enrichment plot and data analysis of the IFN-γ response gene set were determined using Phantasus (39).

### Gene expression analysis.

Lung tissue was frozen on dry ice and stored at –80°C until tissue disruption in 250 μL Dulbecco’s phosphate-buffered saline (DPBS) using a MagNA Lyser (Roche) for 60 seconds at 6 × 10^3^ rpm. Homogenates were centrifuged at 1.5 × 10^4^ *g* for 5 minutes at 4°C, and RNA from 50 μL of supernatant was extracted using the RNeasy Mini Kit (Qiagen) per the manufacturer’s instructions. The TaqMan RNA-to-CT 1-Step Kit (Applied Biosystems) was used to measure mRNA expression. Primers and probes were purchased from Integrated DNA Technologies. Ct values for all target genes were normalized to Ct values of the housekeeping gene *Gapdh*. Fold change in target gene expression was reported as 2^–ΔΔCt^, normalized to the average expression observed in WT mice.

### Lymph node quantitation.

For Evans blue staining, WT and STING N153S mice were anesthetized, and 25 μL of 5% Evans blue dye in PBS was injected into 1 forefoot and 1 hindfoot. Mice were euthanized 15 minutes following dye injection and dissected for quantitation of retroperitoneal and inguinal lymph nodes. Any mesenteric lymph nodes were counted as 1 node.

### Cell isolation.

Spleens and thymi were removed from animals and kept on ice in FACS buffer (DPBS; Gibco, 14190136) supplemented with 5% FBS (Omega Scientific, FB-01). To obtain single-cell suspensions, organs were mechanically dissociated and passed through 70 μm cell strainers and rinsed with ice-cold 20 mL PBS. Red blood cells were lysed in 2 mL ACK lysis buffer (Gibco, A1049201) for 3 minutes, and the cell suspensions were washed once in FACS buffer, counted, and prepared for downstream applications. To isolate bone marrow cells for mixed chimera studies or BMDM differentiation, femurs and tibias were dissected from animals, and the marrow was flushed from bones, followed by debris removal by filtration through 70 μm cell strainers. For T cell isolation, bulk splenic T cells were isolated using the EasySep Mouse T Cell Isolation Kit (StemCell 19751), and purity (routinely ~90%–99%) assessed by flow cytometric analysis of CD3ε expression.

### BMDM generation and cell culture.

To generate BMDMs, 5 × 10^6^ marrow cells were cultured for 8 days at 37°C in 5% CO_2_ in 10 mm petri dishes containing 40 ng/mL recombinant mouse macrophage colony-stimulating factor (R&D Systems, 416-ML) in 10 mL complete DMEM (Gibco, 11965118) supplemented with 10% FBS, 2 mM L-glutaMAX (Gibco, 35050061), 1× nonessential amino acids (Gibco, 11140050), 1 mM sodium pyruvate (Gibco, 11360070), 10 mM HEPES (Gibco, 15630106), and 100 U/mL penicillin and 100 mg/mL streptomycin (Gibco, 10378016). For T cell coculture and costimulation assays, isolated T cells or splenocytes were incubated at 37°C in 5% CO_2_ in complete RPMI 1640 Medium (Gibco, 11879020), containing 20% FBS, all supplements (see above), and 55 μM β-mercaptoethanol (Gibco, 31350010).

### IFN treatment of BMDMs.

BMDMs were treated with 100 U/mL murine IFN-β from *Escherichia coli* (PBL, 12400-1), 10 ng/mL recombinant murine IFN-γ (Peprotech, 12820-1), or 100 ng/mL recombinant murine IFN-λ3 (PBL, 12820-1). Gene expression was quantitated 4 hours later by qRT-PCR. Cell surface marker expression was analyzed 24 hours later by flow cytometry.

### Antibodies.

To confirm the neutralizing capacity of the anti–IFN-γ antibody, 20 μg/mL anti-mouse IFN-γ antibodies (Leinco, I-438) or isotype control antibody (Leinco, I-140) were incubated with recombinant murine IFN-γ (10 ng/mL) for 30 minutes at room temperature and then added to BMDMs and incubated for 4 hours, followed by RNA isolation and ISG expression analysis by qRT-PCR. For anti-mouse antibodies used in flow cytometry and T cell proliferation assays, refer to Supplemental Table 1.

### Antigen-presentation assay.

Antigen-presentation assays were performed as previously described (44). BMDMs were generated as described above. On day 7 after isolation, BMDM growth media was changed to media containing 100ng/mL LPS (MilliporeSigma, L2630) for 24 hours. The following day, BMDMs were harvested and incubated with 100 ng/mL ovalbumin peptide (OVA_257–264_) (MilliporeSigma, S7951) in complete media for 1 hour at 37°C, followed by 3 washes in PBS. OVA-loaded BMDMs were then plated at 25,000 cells per well in T cell media (RPMI 1640, 20% FBS, 1× nonessential amino acids, 1 mM sodium pyruvate, 2 mM L-glutaMax, 100 U/mL penicillin, 100 mg/mL streptomycin, 10 mM HEPES, 55 μM 2-mercaptoethanol) in 96-well round-bottom plates. CD8α^+^ T cells were isolated from OT-I mouse spleens by magnetic bead separation (Miltenyi, 130-104-075) and stained with 1 μM CFSE (BioLegend, 423801) in PBS for 20 minutes at room temperature, followed by 2 washes in T cell media. Next, 100,000 CD8α^+^ T cells were added to the OVA-loaded BMDMs in the 96-well plates and cultured for 3 days. Proliferation of T cells was measured using an LSR II flow cytometer (BD Biosciences) and analyzed by FlowJo v10 software.

### Flow cytometry.

Fc-mediated interactions were blocked by incubating cell suspensions in 1 μg TruStain FcX anti-mouse CD16/32 in 50 μL FACS buffer for 20 minutes on ice. To stain surface antigens, cell suspensions were incubated with fluorescence-conjugated antibodies in 50 μL FACS buffer for 30 minutes on ice and washed 3 times. To assess cellular viability, cell suspensions were washed once in DPBS to remove residual FBS and then stained as indicated with 1:1000 Zombie NIR (BioLegend, 423106), LIVE/DEAD Fixable Aqua (Thermo Fisher, L34957), or LIVE/DEAD Fixable Violet (Thermo Fisher, L34955) in 200 μL DPBS for 15 minutes on ice. For intracellular FoxP3 expression, cells were fixed in FoxP3 Fixation/Permeabilization Buffer (eBiosciences, 00-5523-00) and stained with FoxP3 antibody per the manufacturer’s instructions. For evaluation of apoptosis, surface-antibody labeled cells were washed twice in 1× Annexin V Binding Buffer and stained with Annexin V (BD Biosciences, 556421) per the manufacturer’s instructions. In all experiments, stained cells were acquired on an Attune NxT Flow Cytometer (Thermo Fisher) or LSR II (BD Biosciences), and data analysis was conducted using FlowJo v10 software.

### T cell proliferation and coculture.

One day prior to splenocyte isolation, 24-well tissue culture plates were coated with 1 μg/mL hamster anti-mouse CD3ε and CD28 antibodies in 1 mL DPBS and incubated overnight at 4°C. Following red blood cell lysis, 2 × 10^7^ splenocytes were washed twice in DPBS to remove residual FBS prior to a 15-minute incubation at room temperature in 1 μM carboxyfluorescein succinimidyl ester (CFSE; BioLegend, 423801) in 1.25 mL DPBS. The CFSE-labeling reaction was quenched with 12.5 mL RPMI supplemented with 20% FBS. Antibody-coated wells were washed twice with DPBS and plated with 1 × 10^6^ CFSE-labeled cells and resuspended in a total volume of 1 mL complete RPMI, before a 72-hour incubation at 37°C in 5% CO_2_. Cells were stained with Zombie NIR viability dye and antibodies against CD45, CD3ε, CD4, and CD8α in order to assess cellular division in CD4^+^ and CD8α^+^ T cells. The division index, defined as the average number of cell divisions that a cell in the original population underwent, was determined using the Proliferation Modeling tool in FlowJo. For T cell coculture assays, 1 × 10^5^ isolated CD45.1 WT T cells were incubated with 1 × 10^5^ CD45.2 WT or STING N153S T cells in 200 μL complete RPMI.

### Generation of mixed bone marrow chimeras.

Isolated bone marrow cells from CD45.1/2 STING N153S and CD45.2 *Ifngr1^–/–^* STING N153S mice were mixed at a 1:1 ratio in DPBS without FBS. Seven-week-old Thy1.1 mice were administered a single lethal dose of irradiation (10 Gy) (X-Rad 320 Irradiator, Rad Source Technologies). Next, 5 hours after irradiation, 5 × 10^6^ cells were transplanted via intravenous retro-orbital injection. Donor and recipient animals were all male to mitigate graft-versus-host disease. At 13 weeks of age, spleens were harvested from Thy1.1 mixed bone marrow recipients for FACS analysis of circulating immune cells. Experiments were similarly conducted with bone marrow isolated from CD45.1/2 WT and CD45.2 *Ifngr1^–/–^* animals.

### Statistics.

Statistical significance of the upregulated MSigDB hallmark gene sets was determined in Enrichr (41–43) using Fisher’s exact test with Benjamini-Hochberg correction (FDR = 5%). The adjusted *P* value is reported as a *Q* value in Enrichr (42). Enrichment plot, Fisher’s exact test, and normalized enrichment score of the IFN-γ response gene set were determined using Phantasus (39). Statistical tests are indicated in the figure legends and include Fisher’s exact test with Benjamini-Hochberg correction, Mann-Whitney test, Kruskal-Wallis test, 1-tailed Student’s *t* test, and 1-and 2-way ANOVA. Data are shown as the mean ± SEM. *P* < 0.05 was considered statistically significant. Except for the reanalyzed gene expression data, all analysis was performed using GraphPad Prism9 software.

### Study approval.

This study was performed in accordance with the recommendations in the *Guide for the Care and Use of Laboratory Animals* (National Academies Press, 2011). The protocols were approved by the Institutional Animal Care and Use Committee at the Washington University School of Medicine (assurance no. A3381-01) and the University of Pennsylvania Perelman School of Medicine (assurance no. A3079-01). All efforts were made to minimize animal suffering.

## Author contributions

WAS, CAM, AJL, and FRZ performed experiments and analyzed data. WAS performed tissue harvests and performed many of the T cell studies, including the mixed bone marrow chimeric mouse experiments. CAM performed all of the revision experiments, including coculture studies and BMDM experiments, analyzed data, and carried out manuscript revision. FRZ performed thymocyte analysis. AJL revised the final version of the manuscript and performed qRT-PCR analysis at the revision stage. JJM guided experiments. WAS wrote the initial draft and edited subsequent versions. JJM, CAM, AJL, SP, and FRZ wrote and edited the final version of the manuscript. All authors reviewed the final version of the manuscript. JJM conceived the project and guided all experiments. All authors have critically reviewed the manuscript.

## Supplementary Material

Supplemental data

## Version 1 09/08/2022

Electronic publication
