# Supplementary material for: The IFN-γ receptor promotes immune dysregulation and disease in STING gain-of-function mice
Source: JCI Insight. 2022 Sep 8;7(17):e155250. doi: 10.1172/jci.insight.155250 (PMC9536275; doi:10.1172/jci.insight.155250)
Supplement: Supplemental data [file jciinsight-7-155250-s124.pdf]

SUPPLEMENTAL FIGURES

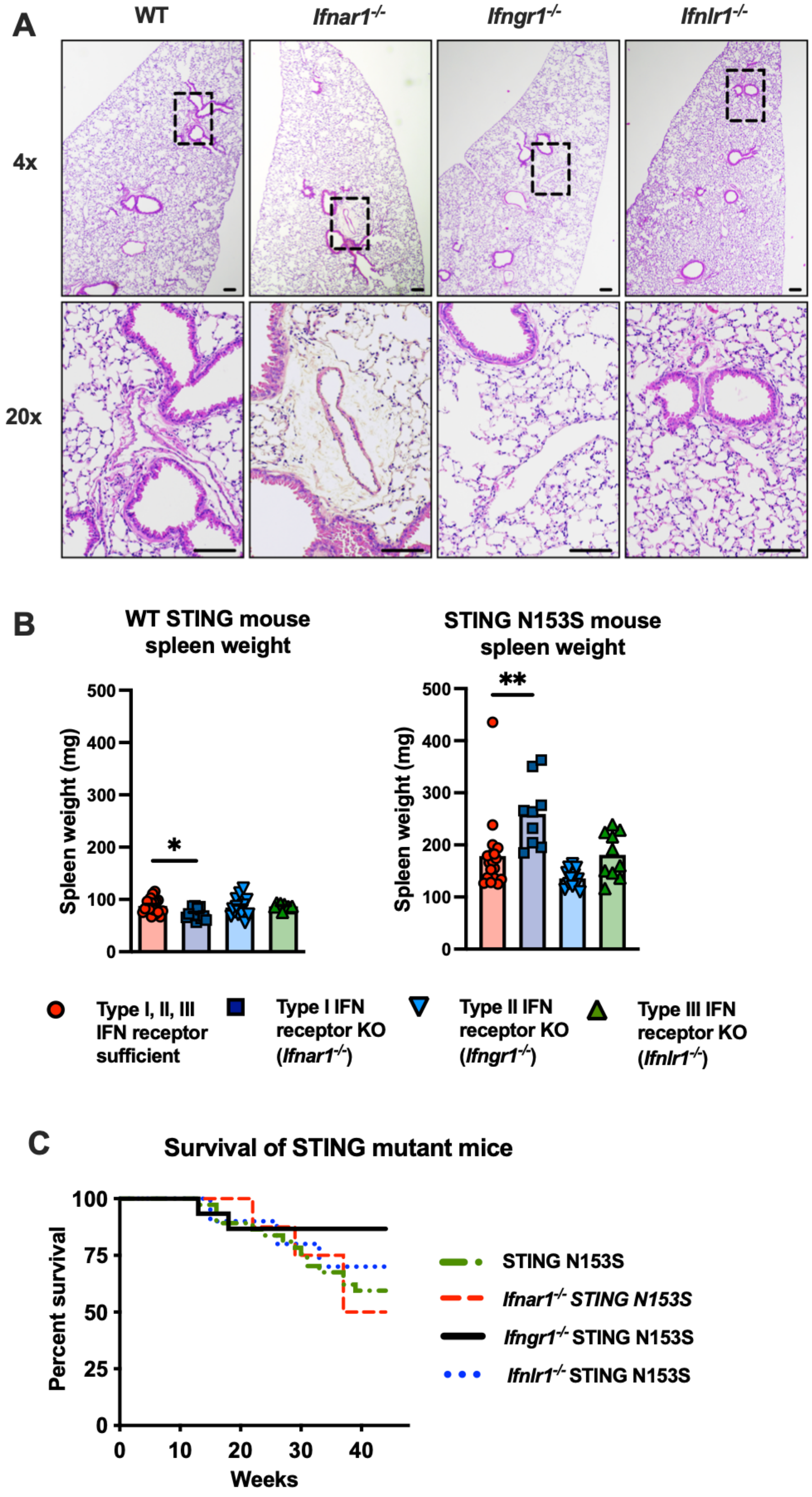

Supplemental Figure 1

Supplemental Figure 1. WT STING-expressing IFN receptor controls and effects of IFN receptor deletion on splenomegaly and survival. (**A)** Representative images of H&E-stained lung sections from 13-16-week-old WT and *Ifngr1*$^{-/-}$ mice. Images are representative of *n* = 10-15 mice per group. Top panel images were taken under low magnification, and bottom panel images were taken under high magnification. Scale bars = 100 μm. **(B)** Spleen weights of 13-16-week-old WT and STING N153S (SAVI) mice, and littermate mice lacking the indicated IFN receptor. Data represent the mean ± SEM of *n* = 9-19 mice per genotype pooled from 3-6 independent experiments. **(C)** Kaplan-Meier survival curve showing percent survival of SAVI mice and those lacking the indicated IFN receptor. Data were obtained from *n* = 35 SAVI mice, *n* = 8 *Ifnar1*$^{-/-}$ SAVI mice, *n* = 15 *Ifngr1*$^{-/-}$ SAVI mice, and *n* = 11 *Ifnlr1*$^{-/-}$ SAVI mice. Data in (B) were analyzed by Kruskal-Wallis test and in (C) by Mantel-Cox Logrank test. *$P < 0.05$, **$P < 0.01$, ***$P < 0.001$, and ****$P < 0.0001$.

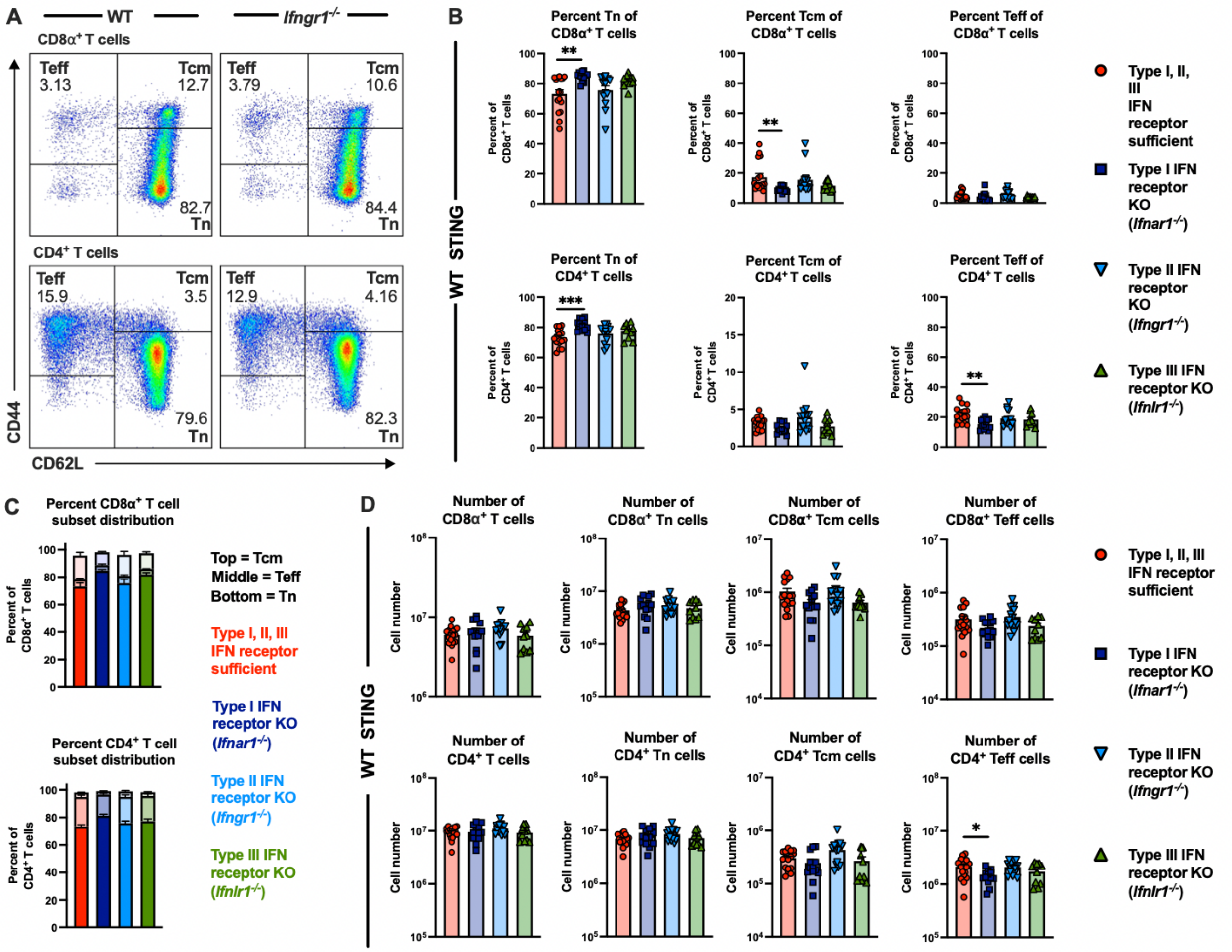

Supplemental Figure 2

Supplemental Figure 2. The type II IFN receptor has no effect on T cell numbers or subsets in WT STING mice. (A) Representative FACS plots of naïve (Tn), central memory (Tcm), and effector memory (Teff) splenic CD8α^{+} (top panels) and CD4^{+} T cells (bottom panels) from WT and *Ifngr1*$^{-/-}$ mice. **(B)** Frequencies of splenic CD8α^{+} and CD4^{+} Tn, Tcm, and Teff cells in WT and *Ifngr1*$^{-/-}$ mice. **(C)** Subset distributions of CD8α^{+} and CD4^{+} T cells in WT and *Ifngr1*$^{-/-}$ mice. **(D)** Numbers of total splenic CD8α^{+} and CD4^{+} T cells, as well as CD8α^{+} and CD4^{+} Tn, Tcm, and Teff subsets, in WT and *Ifngr1*$^{-/-}$ mice. Data in (B and D) represent the mean ± SEM of *n* = 10-17 mice per genotype pooled from 3-6 independent experiments and were analyzed by Kruskal-Wallis

test. Data in (C) represent the mean ± SEM of $n = 4$ mice per genotype pooled from 2 independent experiments. *$P < 0.05$, **$P < 0.1$.

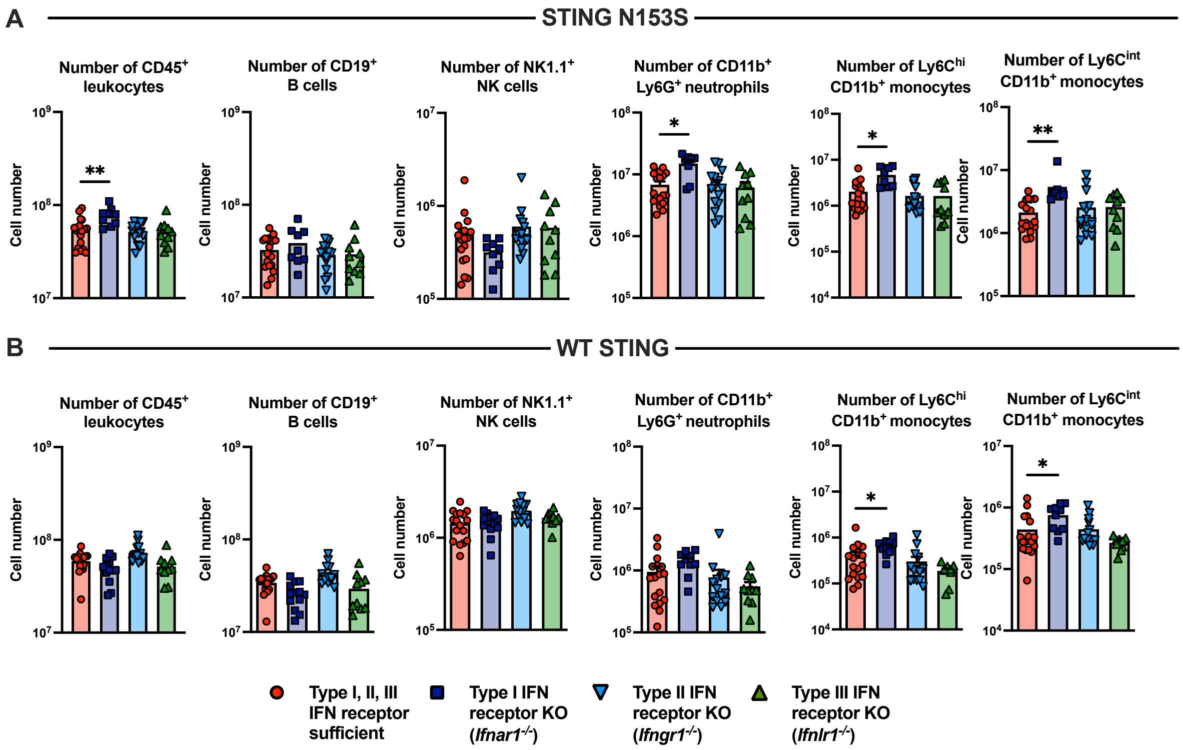

Supplemental Figure 3

Supplemental Figure 3. IFNGR1 does not regulate myeloid cell numbers in SAVI mice. (A-B) Numbers of splenic CD45$^+$ leukocytes, CD19$^+$ B cells, NK1.1$^+$ CD3ε$^-$ natural killer (NK) cells, CD11b$^+$ Ly6G$^+$ neutrophils, LyC^{hi} CD11b$^+$ monocytes, and LyC^{lo} CD11b$^+$ monocytes in STING N153S (SAVI) animals (A) or WT STING littermates (B), including the corresponding IFN receptor knockout mice. Data in (A and B) represent the mean ± SEM of n = 9-18 mice per genotype pooled from 3-6 independent experiments. Data were analyzed by Kruskal-Wallis test. *P < 0.05, **P < 0.01.

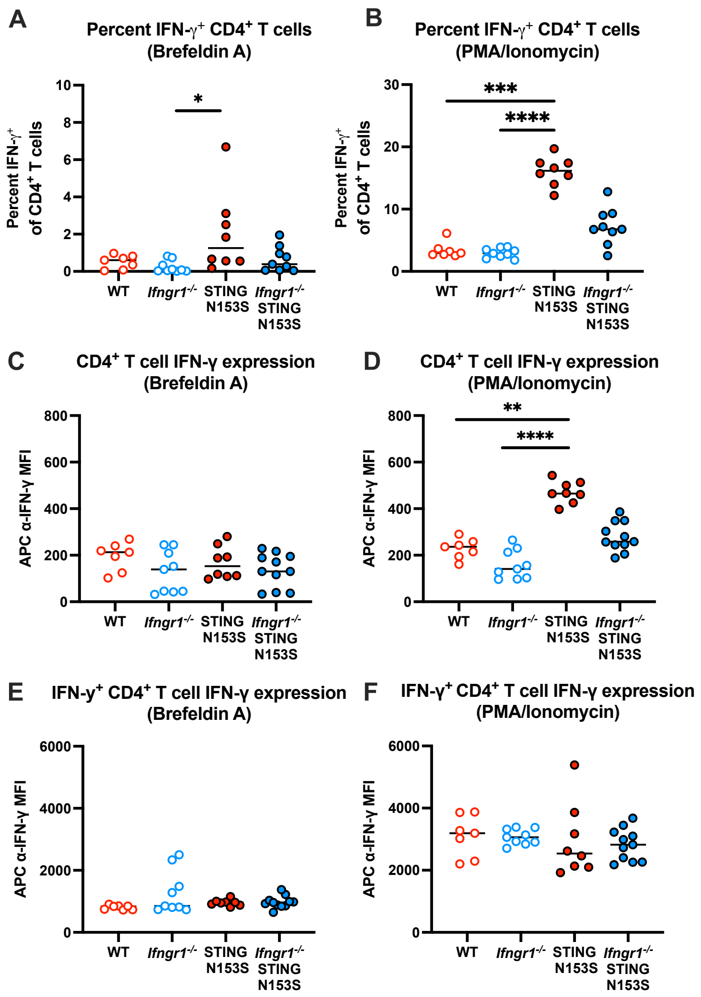

Supplemental Figure 4

Supplemental Figure 4. IFN-γ expression is increased in CD4+ T cells in SAVI mice. (A and B) Percent IFN-γ+ CD4+ T cells from WT, *Ifngr1-/-*, SAVI, and *Ifngr1-/-* SAVI mice after treatment with Brefeldin A (A, C, E) or Brefeldin A plus PMA/Ionomycin (B, D, F). **(C and D)** MFI of IFN-γ expression in all CD4+ T cells from WT, *Ifngr1-/-*, SAVI, and *Ifngr1-/-* SAVI mice after treatment as described in (A and B). **(E and F)** MFI of IFN-γ expression in IFN-γ+ CD4+ T cells from WT, *Ifngr1-/-*, SAVI, and *Ifngr1-/-* SAVI mice treated as described above. Data represent the mean of *n* = 7-9 mice per genotype pooled from 2 independent experiments, and were analyzed by the Kruskal-Wallis test. *$P < 0.05$, **$P < 0.01$, ***$P < 0.001$, and ****$P < 0.0001$.

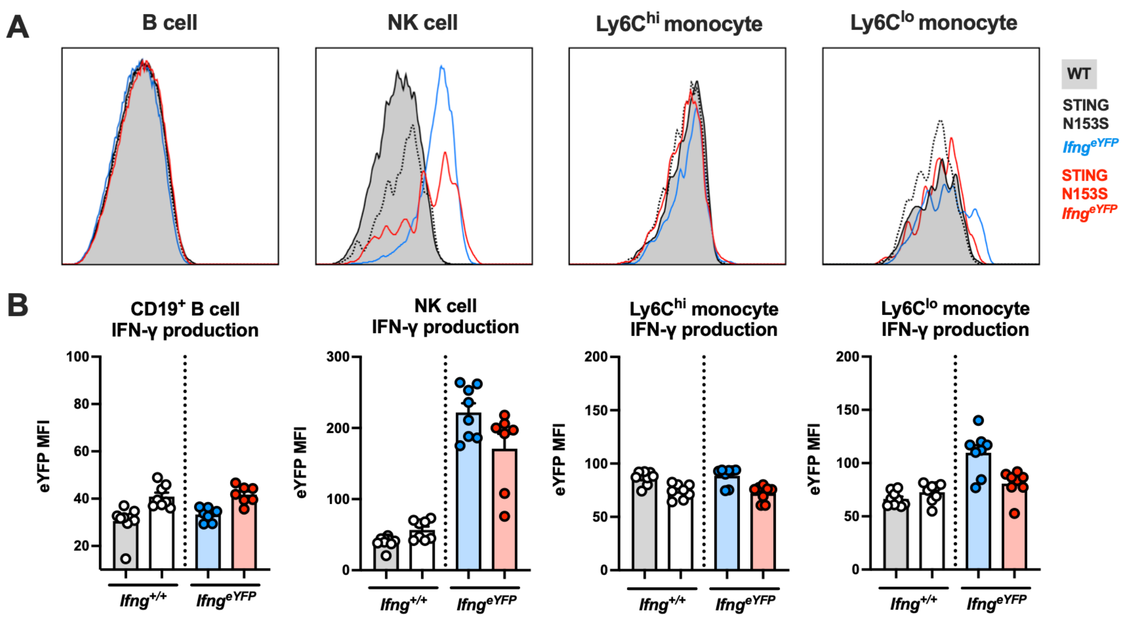

Supplemental Figure 5

Supplemental Figure 5. STING gain-of-function does not enhance IFN-γ expression by B cells, NK cells, or monocytes. (A and B) Representative histogram plots (A) and mean fluorescence intensity (B) of eYFP in splenocytes from WT and STING N153S (SAVI) IFN-γ reporter (*Ifng*^{eYFP}) and non-reporter (*Ifng*$^{+/+}$) control mice. Data represent the mean of *n* = 7-9 mice pooled from two-independent experiments. Data were analyzed by Mann-Whitney test.

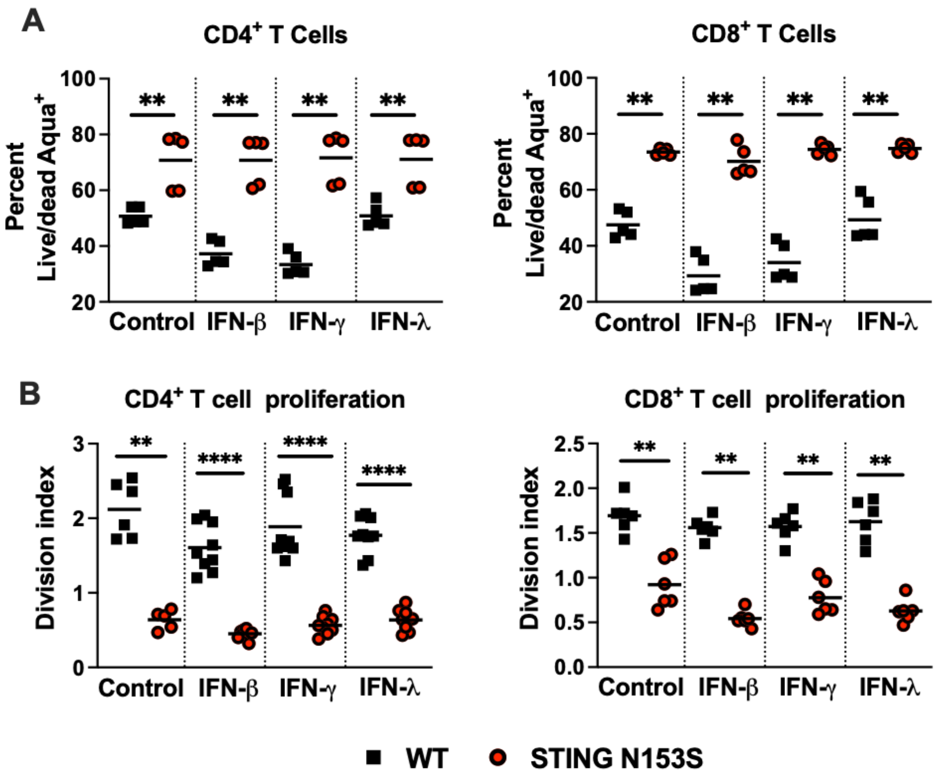

Supplemental Figure 6

Supplemental Figure 6. Treatment with type I, type II, or type III IFN does not alter SAVI mouse T-cell survival or proliferation. (A) Percent Live/dead$^+$ WT and SAVI CD4$^+$ (left panel) and CD8α$^+$ (right panel) T cells after 24 hours in the presence of type I, II, or III IFNs. Data represent the mean of n = 6 samples per group from 2 independent experiments. **(B)** Average number of cell divisions of CD4$^+$ (left panel) and CD8α$^+$ (right panel) T cells from WT and SAVI mice, as determined by CFSE dilution. T cells, pre-treated with the indicated IFN for 4 hours, were stimulated with antibodies against CD3ε and CD28 in the presence of the indicated IFN for 3 days prior to evaluation by flow cytometry. Data represent the mean of n = 6-9 samples per group from 2 independent experiments. Data in were analyzed by Mann-Whitney test. *P < 0.05, **P < 0.01, ***P < 0.001, and ****P < 0.0001.

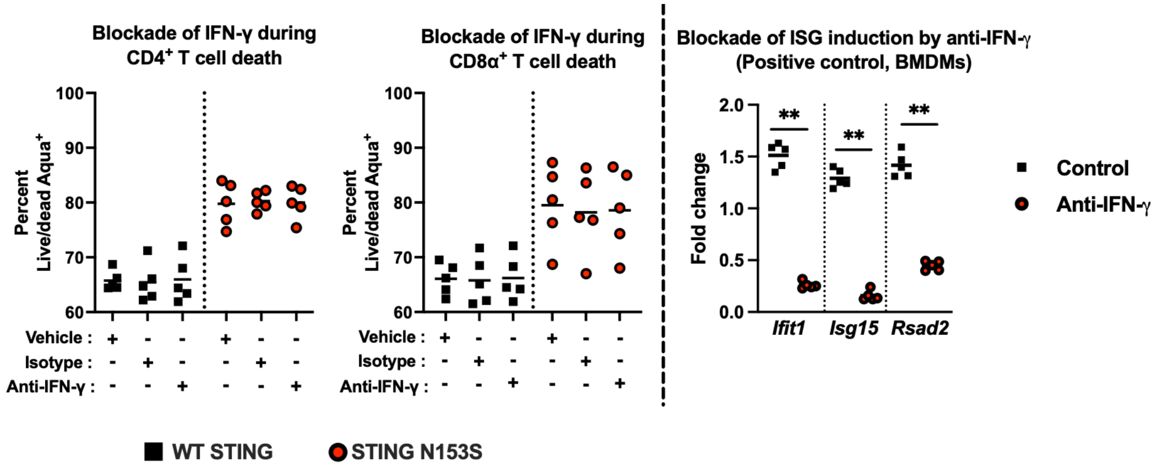

Supplemental Figure 7

Supplemental Figure 7. Treatment with an IFN-γ blocking antibody has no effect on SAVI mouse T-cell death. Percent live/dead CD4$^+$ and CD8α$^+$ T cells from WT and SAVI mice upon treatment with an IFN-γ blocking antibody, compared to vehicle, isotype, and positive controls. Cells were incubated with recombinant murine IFN-γ for 24 hours and then assessed for viability via Live/dead stain. Data represent the mean of n = 5 samples pooled from two independent experiments and were analyzed by Kruskal-Wallis test (left panels) and Mann-Whitney test (right panel). *P < 0.05, **P < 0.01.

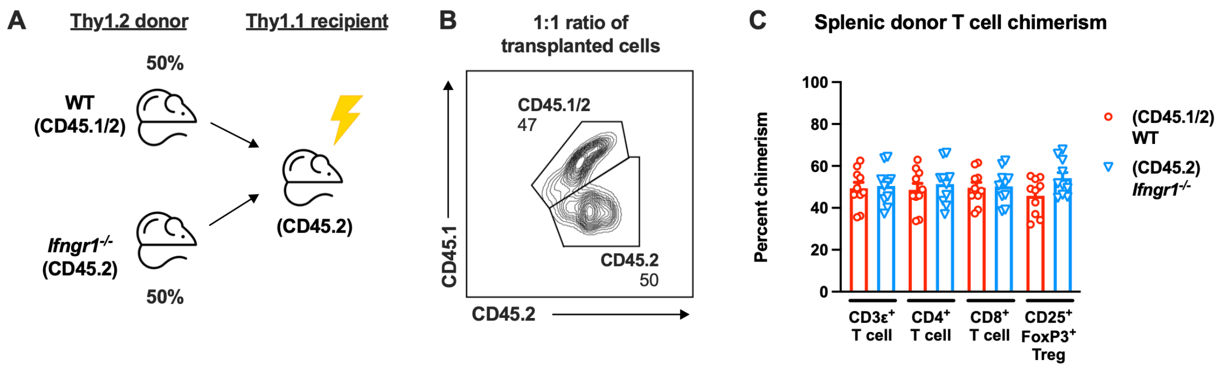

Supplemental Figure 8

Supplemental Figure 8. In mixed bone marrow chimeric mice, WT and *Ifngr1$^{-/-}$* bone marrow mixed 1:1 results in equivalent chimerism of T cells. (A) Diagram of the strategy used to generate Thy1.1 mixed bone marrow chimeric mice. **(B)** Representative FACS plot of the CD45.1/2 WT and CD45.2 *Ifngr1$^{-/-}$* bone marrow cells, which were transferred at a ~1:1 ratio. **(C)** Percentages of circulating CD45.1/2 SAVI and CD45.2 *Ifngr1$^{-/-}$* SAVI CD3ε^{+}, CD4^{+}, CD8α^{+} T cells, and CD25^{+} FoxP3^{+} Tregs in irradiated Thy1.1^{+} animals. Data in (C) represent the mean $\pm$ SEM of the indicated T-cell populations isolated from the spleens of n = 10 Thy1.1^{+} chimeric mice from two independent experiments. Data were analyzed by Mann-Whitney test.

Target	Target Species	Conjugate	Company	Isotype	Clone	Catalog Number	Concentration	Assay
CD16/CD32	ms	--	BioLegend	Rat IgG2a, λ	93	101320	1:50	FcR block
CD45	ms	BV510	BioLegend	Rat IgG2b, κ	30-F11	103138	1:200	Flow
CD45	ms	BV605	BioLegend	Rat IgG2b, κ	30-F11	103140	1:200	Flow
CD45.1	ms	APC-Cy7	BioLegend	Mouse (A.SW) IgG2a, κ	A20	110752	1:200	Flow
CD45.2	ms	FITC	BioLegend	Mouse (SJL) IgG2a, κ	104	109806	1:200	Flow
CD3ε	ms	BV510	BioLegend	Armenian Hamster IgG	145-2C11	100353	1:100	Flow
CD3ε	ms	APC	BioLegend	Armenian Hamster IgG	145-2C11	152306	1:100	Flow
CD19	ms	FITC	BioLegend	Rat IgG2a, κ	6D5	115506	1:100	Flow
NK1.1	ms	APC-Cy7	BioLegend	Mouse IgG2a, κ	PK136	108724	1:100	Flow
CD4	ms	BV421	BioLegend	Rat IgG2b, κ	GK1.5	100438	1:200	Flow
CD4	ms	BV605	BioLegend	Rat IgG2b, κ	GK1.5	100451	1:200	Flow
CD8α	ms	PerCP-Cy5.5	BioLegend	Rat IgG2a, κ	53-6.7	100734	1:100	Flow
CD8α	ms	BV785	BioLegend	Rat IgG2a, κ	53-6.7	100750	1:100	Flow
CD44	ms/hu	APC	BioLegend	Rat IgG2b, κ	IM7	103012	1:200	Flow
CD44	ms/hu	BV605	BioLegend	Rat IgG2b, κ	IM7	103049	1:200	Flow
CD62L	ms	PE	BioLegend	Rat IgG2a, κ	MEL-14	104408	1:200	Flow
CD11b	ms/hu	BV510	BioLegend	Rat IgG2b, κ	M1/70	101263	1:100	Flow
CD11c	ms	AF488	BioLegend	Armenian Hamster IgG	N418	117311	1:200	Flow
Ly6C	ms	BV421	BioLegend	Rat IgG2c, κ	HK1.4	128032	1:200	Flow
Ly6G	ms	PerCP-Cy5.5	BioLegend	Rat IgG2a, κ	1A8	127616	1:200	Flow
I-A/I-E	ms	PE	BioLegend	Rat IgG2b, κ	M5/114.15.2	107608	1:200	Flow
I-A/I-E	ms	APC-Cy7	BioLegend	Rat IgG2b, κ	M5/114.15.2	107628	1:200	Flow
F4/80	ms	APC	BioLegend	Rat IgG2a, κ	BM8	123116	1:500	Flow
CD69	ms	PE	BioLegend	Armenian Hamster IgG	H1.2F3	104507	1:500	Flow
CD86	ms	BV785	BioLegend	Rat IgG2a, κ	GL-1	105043	1:500	Flow
CD25	ms	FITC	BioLegend	Rat IgG1, λ	PC61	102036	1:100	Flow
CD25	ms	BV605	BioLegend	Rat IgG1, λ	PC61	102006	1:100	Flow
FoxP3	ms/hu*	PE	eBiosciences	Rat IgG2a, κ	FJK-16s	12-5773-82	1:20	Flow
CD3ε	ms	--	BioLegend	Armenian Hamster IgG	145-2C11	100302	0.1 or 1ug/ml	T cell activation
CD28	ms	--	BioLegend	Syrian Hamster IgG	37.51	102102	0.1 or 1ug/ml	T cell activation

Supplemental Table 1. List of antibodies used in flow cytometry, T-cell proliferation, and T-cell co-culture studies. Abbreviations are: ms = mouse; hu = human; ha = hamster; * = reported cross-reactivity.
